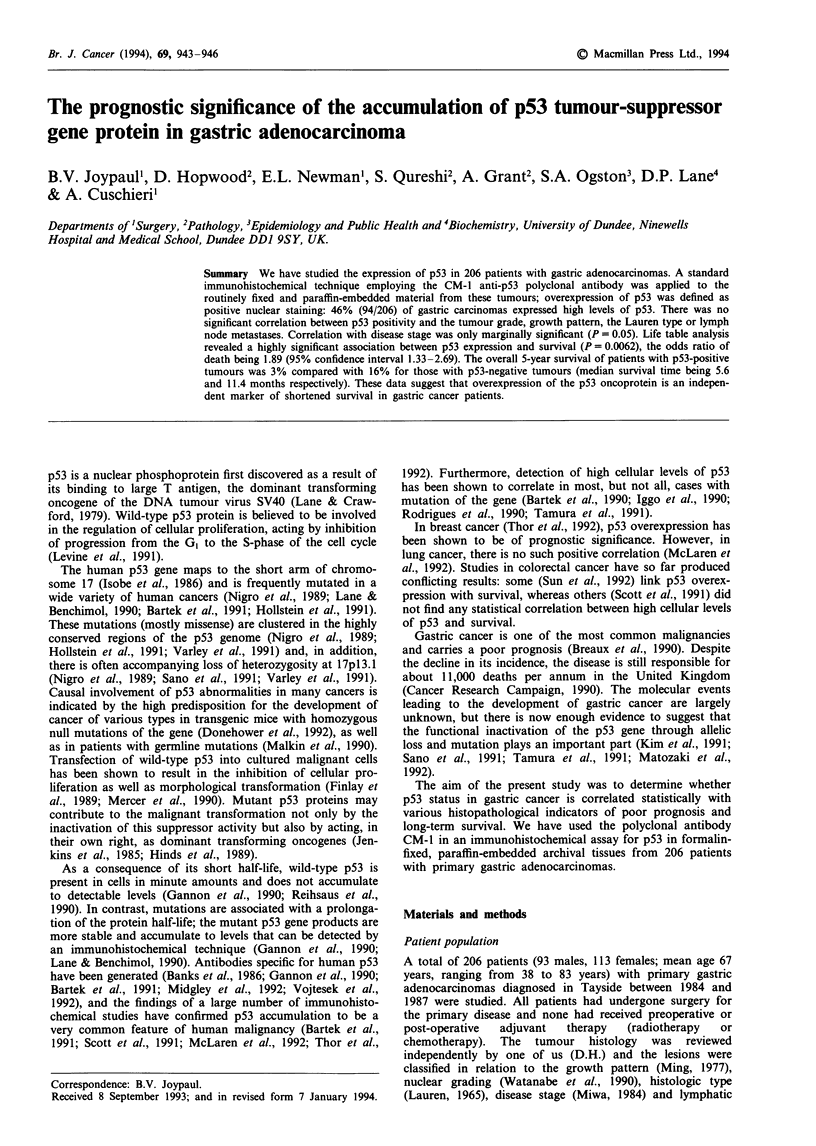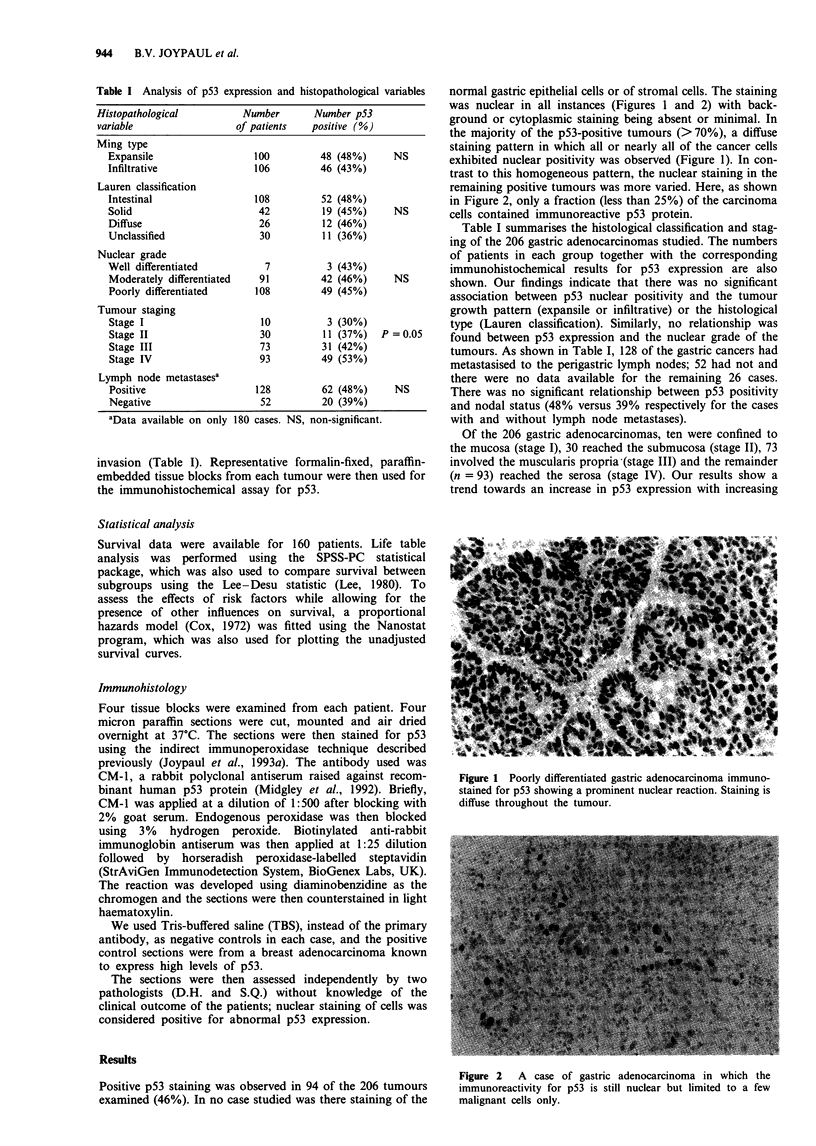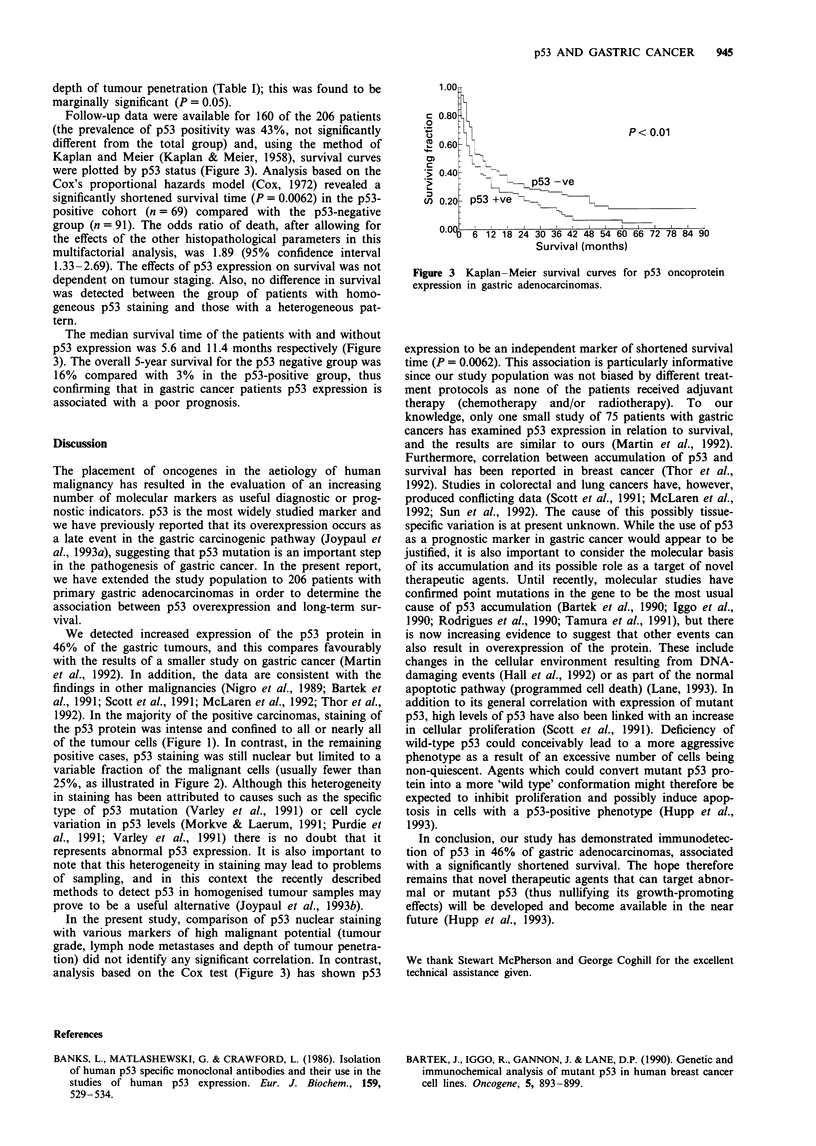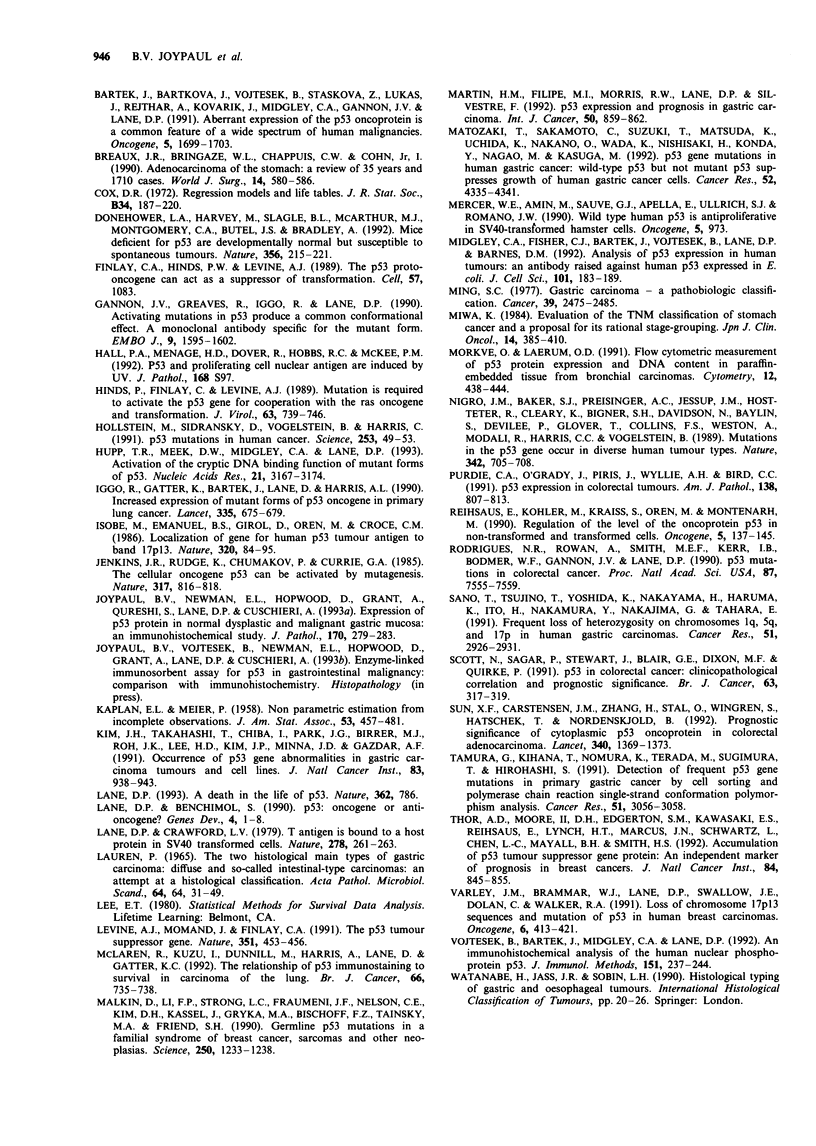# The prognostic significance of the accumulation of p53 tumour-suppressor gene protein in gastric adenocarcinoma.

**DOI:** 10.1038/bjc.1994.182

**Published:** 1994-05

**Authors:** B. V. Joypaul, D. Hopwood, E. L. Newman, S. Qureshi, A. Grant, S. A. Ogston, D. P. Lane, A. Cuschieri

**Affiliations:** Department of Surgery, University of Dundee, Ninewells Hospital and Medical School, UK.

## Abstract

**Images:**


*Br. J. Cancer* (1994), **69**, 943–946 © Macmillan Press Ltd., 1994

### The prognostic significance of the accumulation of p53 tumour-suppressor gene protein in gastric adenocarcinoma

B.V. Joypaul[1], D. Hopwood[2], E.L. Newman[1], S. Qureshi[2], A. Grant[2], S.A. Ogston[3], D.P. Lane[4] & A. Cuschieri[1]

*Departments of [1]Surgery, [2]Pathology, [3]Epidemiology and Public Health and [4]Biochemistry, University of Dundee, Ninewells Hospital and Medical School, Dundee DD1 9SY, UK.*

**Summary** We have studied the expression of p53 in 206 patients with gastric adenocarcinomas. A standard immunohistochemical technique employing the CM-1 anti-p53 polyclonal antibody was applied to the routinely fixed and paraffin-embedded material from these tumours; overexpression of p53 was defined as positive nuclear staining: 46% (94/206) of gastric carcinomas expressed high levels of p53. There was no significant correlation between p53 positivity and the tumour grade, growth pattern, the Lauren type or lymph node metastases. Correlation with disease stage was only marginally significant ($P = 0.05$). Life table analysis revealed a highly significant association between p53 expression and survival ($P = 0.0062$), the odds ratio of death being 1.89 (95% confidence interval 1.33–2.69). The overall 5-year survival of patients with p53-positive tumours was 3% compared with 16% for those with p53-negative tumours (median survival time being 5.6 and 11.4 months respectively). These data suggest that overexpression of the p53 oncoprotein is an independent marker of shortened survival in gastric cancer patients.

p53 is a nuclear phosphoprotein first discovered as a result of its binding to large T antigen, the dominant transforming oncogene of the DNA tumour virus SV40 (Lane & Crawford, 1979). Wild-type p53 protein is believed to be involved in the regulation of cellular proliferation, acting by inhibition of progression from the $G_1$ to the S-phase of the cell cycle (Levine *et al.*, 1991).

The human p53 gene maps to the short arm of chromosome 17 (Isobe *et al.*, 1986) and is frequently mutated in a wide variety of human cancers (Nigro *et al.*, 1989; Lane & Benchimol, 1990; Bartek *et al.*, 1991; Hollstein *et al.*, 1991). These mutations (mostly missense) are clustered in the highly conserved regions of the p53 genome (Nigro *et al.*, 1989; Hollstein *et al.*, 1991; Varley *et al.*, 1991) and, in addition, there is often accompanying loss of heterozygosity at 17p13.1 (Nigro *et al.*, 1989; Sano *et al.*, 1991; Varley *et al.*, 1991). Causal involvement of p53 abnormalities in many cancers is indicated by the high predisposition for the development of cancer of various types in transgenic mice with homozygous null mutations of the gene (Donehower *et al.*, 1992), as well as in patients with germline mutations (Malkin *et al.*, 1990). Transfection of wild-type p53 into cultured malignant cells has been shown to result in the inhibition of cellular proliferation as well as morphological transformation (Finlay *et al.*, 1989; Mercer *et al.*, 1990). Mutant p53 proteins may contribute to the malignant transformation not only by the inactivation of this suppressor activity but also by acting, in their own right, as dominant transforming oncogenes (Jenkins *et al.*, 1985; Hinds *et al.*, 1989).

As a consequence of its short half-life, wild-type p53 is present in cells in minute amounts and does not accumulate to detectable levels (Gannon *et al.*, 1990; Reihsaus *et al.*, 1990). In contrast, mutations are associated with a prolongation of the protein half-life; the mutant p53 gene products are more stable and accumulate to levels that can be detected by an immunohistochemical technique (Gannon *et al.*, 1990; Lane & Benchimol, 1990). Antibodies specific for human p53 have been generated (Banks *et al.*, 1986; Gannon *et al.*, 1990; Bartek *et al.*, 1991; Midgley *et al.*, 1992; Vojtesek *et al.*, 1992), and the findings of a large number of immunohistochemical studies have confirmed p53 accumulation to be a very common feature of human malignancy (Bartek *et al.*, 1991; Scott *et al.*, 1991; McLaren *et al.*, 1992; Thor *et al.*, 1992). Furthermore, detection of high cellular levels of p53 has been shown to correlate in most, but not all, cases with mutation of the gene (Bartek *et al.*, 1990; Iggo *et al.*, 1990; Rodrigues *et al.*, 1990; Tamura *et al.*, 1991).

In breast cancer (Thor *et al.*, 1992), p53 overexpression has been shown to be of prognostic significance. However, in lung cancer, there is no such positive correlation (McLaren *et al.*, 1992). Studies in colorectal cancer have so far produced conflicting results: some (Sun *et al.*, 1992) link p53 overexpression with survival, whereas others (Scott *et al.*, 1991) did not find any statistical correlation between high cellular levels of p53 and survival.

Gastric cancer is one of the most common malignancies and carries a poor prognosis (Breaux *et al.*, 1990). Despite the decline in its incidence, the disease is still responsible for about 11,000 deaths per annum in the United Kingdom (Cancer Research Campaign, 1990). The molecular events leading to the development of gastric cancer are largely unknown, but there is now enough evidence to suggest that the functional inactivation of the p53 gene through allelic loss and mutation plays an important part (Kim *et al.*, 1991; Sano *et al.*, 1991; Tamura *et al.*, 1991; Matozaki *et al.*, 1992).

The aim of the present study was to determine whether p53 status in gastric cancer is correlated statistically with various histopathological indicators of poor prognosis and long-term survival. We have used the polyclonal antibody CM-1 in an immunohistochemical assay for p53 in formalin-fixed, paraffin-embedded archival tissues from 206 patients with primary gastric adenocarcinomas.

### Materials and methods

### *Patient population*

A total of 206 patients (93 males, 113 females; mean age 67 years, ranging from 38 to 83 years) with primary gastric adenocarcinomas diagnosed in Tayside between 1984 and 1987 were studied. All patients had undergone surgery for the primary disease and none had received preoperative or post-operative adjuvant therapy (radiotherapy or chemotherapy). The tumour histology was reviewed independently by one of us (D.H.) and the lesions were classified in relation to the growth pattern (Ming, 1977), nuclear grading (Watanabe *et al.*, 1990), histologic type (Lauren, 1965), disease stage (Miwa, 1984) and lymphatic

Correspondence: B.V. Joypaul.
Received 8 September 1993; and in revised form 7 January 1994.

**Table I** Analysis of p53 expression and histopathological variables

| *Histopathological variable* | *Number of patients* | *Number p53 positive (%)* | |
|---|---|---|---|
| Ming type | | | |
| Expansile | 100 | 48 (48%) | NS |
| Infiltrative | 106 | 46 (43%) | |
| Lauren classification | | | |
| Intestinal | 108 | 52 (48%) | |
| Solid | 42 | 19 (45%) | NS |
| Diffuse | 26 | 12 (46%) | |
| Unclassified | 30 | 11 (36%) | |
| Nuclear grade | | | |
| Well differentiated | 7 | 3 (43%) | |
| Moderately differentiated | 91 | 42 (46%) | NS |
| Poorly differentiated | 108 | 49 (45%) | |
| Tumour staging | | | |
| Stage I | 10 | 3 (30%) | |
| Stage II | 30 | 11 (37%) | $P = 0.05$ |
| Stage III | 73 | 31 (42%) | |
| Stage IV | 93 | 49 (53%) | |
| Lymph node metastases[a] | | | |
| Positive | 128 | 62 (48%) | NS |
| Negative | 52 | 20 (39%) | |

[a]Data available on only 180 cases. NS, non-significant.

invasion (Table I). Representative formalin-fixed, paraffin-embedded tissue blocks from each tumour were then used for the immunohistochemical assay for p53.

*Statistical analysis*

Survival data were available for 160 patients. Life table analysis was performed using the SPSS-PC statistical package, which was also used to compare survival between subgroups using the Lee–Desu statistic (Lee, 1980). To assess the effects of risk factors while allowing for the presence of other influences on survival, a proportional hazards model (Cox, 1972) was fitted using the Nanostat program, which was also used for plotting the unadjusted survival curves.

*Immunohistology*

Four tissue blocks were examined from each patient. Four micron paraffin sections were cut, mounted and air dried overnight at 37°C. The sections were then stained for p53 using the indirect immunoperoxidase technique described previously (Joypaul *et al.*, 1993*a*). The antibody used was CM-1, a rabbit polyclonal antiserum raised against recombinant human p53 protein (Midgley *et al.*, 1992). Briefly, CM-1 was applied at a dilution of 1:500 after blocking with 2% goat serum. Endogenous peroxidase was then blocked using 3% hydrogen peroxide. Biotinylated anti-rabbit immunoglobin antiserum was then applied at 1:25 dilution followed by horseradish peroxidase-labelled steptavidin (StrAviGen Immunodetection System, BioGenex Labs, UK). The reaction was developed using diaminobenzidine as the chromogen and the sections were then counterstained in light haematoxylin.

We used Tris-buffered saline (TBS), instead of the primary antibody, as negative controls in each case, and the positive control sections were from a breast adenocarcinoma known to express high levels of p53.

The sections were then assessed independently by two pathologists (D.H. and S.Q.) without knowledge of the clinical outcome of the patients; nuclear staining of cells was considered positive for abnormal p53 expression.

### Results

Positive p53 staining was observed in 94 of the 206 tumours examined (46%). In no case studied was there staining of the normal gastric epithelial cells or of stromal cells. The staining was nuclear in all instances (Figures 1 and 2) with background or cytoplasmic staining being absent or minimal. In the majority of the p53-positive tumours (>70%), a diffuse staining pattern in which all or nearly all of the cancer cells exhibited nuclear positivity was observed (Figure 1). In contrast to this homogeneous pattern, the nuclear staining in the remaining positive tumours was more varied. Here, as shown in Figure 2, only a fraction (less than 25%) of the carcinoma cells contained immunoreactive p53 protein.

Table I summarises the histological classification and staging of the 206 gastric adenocarcinomas studied. The numbers of patients in each group together with the corresponding immunohistochemical results for p53 expression are also shown. Our findings indicate that there was no significant association between p53 nuclear positivity and the tumour growth pattern (expansile or infiltrative) or the histological type (Lauren classification). Similarly, no relationship was found between p53 expression and the nuclear grade of the tumours. As shown in Table I, 128 of the gastric cancers had metastasised to the perigastric lymph nodes; 52 had not and there were no data available for the remaining 26 cases. There was no significant relationship between p53 positivity and nodal status (48% versus 39% respectively for the cases with and without lymph node metastases).

Of the 206 gastric adenocarcinomas, ten were confined to the mucosa (stage I), 30 reached the submucosa (stage II), 73 involved the muscularis propria (stage III) and the remainder ($n = 93$) reached the serosa (stage IV). Our results show a trend towards an increase in p53 expression with increasing

**Figure 1** Poorly differentiated gastric adenocarcinoma immunostained for p53 showing a prominent nuclear reaction. Staining is diffuse throughout the tumour.

**Figure 2** A case of gastric adenocarcinoma in which the immunoreactivity for p53 is still nuclear but limited to a few malignant cells only.

depth of tumour penetration (Table I); this was found to be marginally significant ($P = 0.05$).

Follow-up data were available for 160 of the 206 patients (the prevalence of p53 positivity was 43%, not significantly different from the total group) and, using the method of Kaplan and Meier (Kaplan & Meier, 1958), survival curves were plotted by p53 status (Figure 3). Analysis based on the Cox's proportional hazards model (Cox, 1972) revealed a significantly shortened survival time ($P = 0.0062$) in the p53-positive cohort ($n = 69$) compared with the p53-negative group ($n = 91$). The odds ratio of death, after allowing for the effects of the other histopathological parameters in this multifactorial analysis, was 1.89 (95% confidence interval 1.33–2.69). The effects of p53 expression on survival was not dependent on tumour staging. Also, no difference in survival was detected between the group of patients with homogeneous p53 staining and those with a heterogeneous pattern.

The median survival time of the patients with and without p53 expression was 5.6 and 11.4 months respectively (Figure 3). The overall 5-year survival for the p53 negative group was 16% compared with 3% in the p53-positive group, thus confirming that in gastric cancer patients p53 expression is associated with a poor prognosis.

**Figure 3** Kaplan–Meier survival curves for p53 oncoprotein expression in gastric adenocarcinomas.

### Discussion

The placement of oncogenes in the aetiology of human malignancy has resulted in the evaluation of an increasing number of molecular markers as useful diagnostic or prognostic indicators. p53 is the most widely studied marker and we have previously reported that its overexpression occurs as a late event in the gastric carcinogenic pathway (Joypaul *et al.*, 1993*a*), suggesting that p53 mutation is an important step in the pathogenesis of gastric cancer. In the present report, we have extended the study population to 206 patients with primary gastric adenocarcinomas in order to determine the association between p53 overexpression and long-term survival.

We detected increased expression of the p53 protein in 46% of the gastric tumours, and this compares favourably with the results of a smaller study on gastric cancer (Martin *et al.*, 1992). In addition, the data are consistent with the findings in other malignancies (Nigro *et al.*, 1989; Bartek *et al.*, 1991; Scott *et al.*, 1991; McLaren *et al.*, 1992; Thor *et al.*, 1992). In the majority of the positive carcinomas, staining of the p53 protein was intense and confined to all or nearly all of the tumour cells (Figure 1). In contrast, in the remaining positive cases, p53 staining was still nuclear but limited to a variable fraction of the malignant cells (usually fewer than 25%, as illustrated in Figure 2). Although this heterogeneity in staining has been attributed to causes such as the specific type of p53 mutation (Varley *et al.*, 1991) or cell cycle variation in p53 levels (Morkve & Laerum, 1991; Purdie *et al.*, 1991; Varley *et al.*, 1991) there is no doubt that it represents abnormal p53 expression. It is also important to note that this heterogeneity in staining may lead to problems of sampling, and in this context the recently described methods to detect p53 in homogenised tumour samples may prove to be a useful alternative (Joypaul *et al.*, 1993*b*).

In the present study, comparison of p53 nuclear staining with various markers of high malignant potential (tumour grade, lymph node metastases and depth of tumour penetration) did not identify any significant correlation. In contrast, analysis based on the Cox test (Figure 3) has shown p53 expression to be an independent marker of shortened survival time ($P = 0.0062$). This association is particularly informative since our study population was not biased by different treatment protocols as none of the patients received adjuvant therapy (chemotherapy and/or radiotherapy). To our knowledge, only one small study of 75 patients with gastric cancers has examined p53 expression in relation to survival, and the results are similar to ours (Martin *et al.*, 1992). Furthermore, correlation between accumulation of p53 and survival has been reported in breast cancer (Thor *et al.*, 1992). Studies in colorectal and lung cancers have, however, produced conflicting data (Scott *et al.*, 1991; McLaren *et al.*, 1992; Sun *et al.*, 1992). The cause of this possibly tissue-specific variation is at present unknown. While the use of p53 as a prognostic marker in gastric cancer would appear to be justified, it is also important to consider the molecular basis of its accumulation and its possible role as a target of novel therapeutic agents. Until recently, molecular studies have confirmed point mutations in the gene to be the most usual cause of p53 accumulation (Bartek *et al.*, 1990; Iggo *et al.*, 1990; Rodrigues *et al.*, 1990; Tamura *et al.*, 1991), but there is now increasing evidence to suggest that other events can also result in overexpression of the protein. These include changes in the cellular environment resulting from DNA-damaging events (Hall *et al.*, 1992) or as part of the normal apoptotic pathway (programmed cell death) (Lane, 1993). In addition to its general correlation with expression of mutant p53, high levels of p53 have also been linked with an increase in cellular proliferation (Scott *et al.*, 1991). Deficiency of wild-type p53 could conceivably lead to a more aggressive phenotype as a result of an excessive number of cells being non-quiescent. Agents which could convert mutant p53 protein into a more 'wild type' conformation might therefore be expected to inhibit proliferation and possibly induce apoptosis in cells with a p53-positive phenotype (Hupp *et al.*, 1993).

In conclusion, our study has demonstrated immunodetection of p53 in 46% of gastric adenocarcinomas, associated with a significantly shortened survival. The hope therefore remains that novel therapeutic agents that can target abnormal or mutant p53 (thus nullifying its growth-promoting effects) will be developed and become available in the near future (Hupp *et al.*, 1993).

We thank Stewart McPherson and George Coghill for the excellent technical assistance given.

### References

BANKS, L., MATLASHEWSKI, G. & CRAWFORD, L. (1986). Isolation of human p53 specific monoclonal antibodies and their use in the studies of human p53 expression. *Eur. J. Biochem.*, **159**, 529–534.

BARTEK, J., IGGO, R., GANNON, J. & LANE, D.P. (1990). Genetic and immunochemical analysis of mutant p53 in human breast cancer cell lines. *Oncogene*, **5**, 893–899.

BARTEK, J., BARTKOVA, J., VOJTESEK, B., STASKOVA, Z., LUKAS, J., REJTHAR, A., KOVARIK, J., MIDGLEY, C.A., GANNON, J.V. & LANE, D.P. (1991). Aberrant expression of the p53 oncoprotein is a common feature of a wide spectrum of human malignancies. *Oncogene*, **5**, 1699-1703.

BREAUX, J.R., BRINGAZE, W.L., CHAPPUIS, C.W. & COHN, Jr, I. (1990). Adenocarcinoma of the stomach: a review of 35 years and 1710 cases. *World J. Surg.*, **14**, 580-586.

COX, D.R. (1972). Regression models and life tables. *J. R. Stat. Soc.*, **B34**, 187-220.

DONEHOWER, L.A., HARVEY, M., SLAGLE, B.L., MCARTHUR, M.J., MONTGOMERY, C.A., BUTEL, J.S. & BRADLEY, A. (1992). Mice deficient for p53 are developmentally normal but susceptible to spontaneous tumours. *Nature*, **356**, 215-221.

FINLAY, C.A., HINDS, P.W. & LEVINE, A.J. (1989). The p53 proto-oncogene can act as a suppressor of transformation. *Cell*, **57**, 1083.

GANNON, J.V., GREAVES, R., IGGO, R. & LANE, D.P. (1990). Activating mutations in p53 produce a common conformational effect. A monoclonal antibody specific for the mutant form. *EMBO J.*, **9**, 1595-1602.

HALL, P.A., MENAGE, H.D., DOVER, R., HOBBS, R.C. & MCKEE, P.M. (1992). P53 and proliferating cell nuclear antigen are induced by UV. *J. Pathol.*, **168** S97.

HINDS, P., FINLAY, C. & LEVINE, A.J. (1989). Mutation is required to activate the p53 gene for cooperation with the ras oncogene and transformation. *J. Virol.*, **63**, 739-746.

HOLLSTEIN, M., SIDRANSKY, D., VOGELSTEIN, B. & HARRIS, C. (1991). p53 mutations in human cancer. *Science*, **253**, 49-53.

HUPP, T.R., MEEK, D.W., MIDGLEY, C.A. & LANE, D.P. (1993). Activation of the cryptic DNA binding function of mutant forms of p53. *Nucleic Acids Res.*, **21**, 3167-3174.

IGGO, R., GATTER, K., BARTEK, J., LANE, D. & HARRIS, A.L. (1990). Increased expression of mutant forms of p53 oncogene in primary lung cancer. *Lancet*, **335**, 675-679.

ISOBE, M., EMANUEL, B.S., GIROL, D., OREN, M. & CROCE, C.M. (1986). Localization of gene for human p53 tumour antigen to band 17p13. *Nature*, **320**, 84-95.

JENKINS, J.R., RUDGE, K., CHUMAKOV, P. & CURRIE, G.A. (1985). The cellular oncogene p53 can be activated by mutagenesis. *Nature*, **317**, 816-818.

JOYPAUL, B.V., NEWMAN, E.L., HOPWOOD, D., GRANT, A., QURESHI, S., LANE, D.P. & CUSCHIERI, A. (1993*a*). Expression of p53 protein in normal dysplastic and malignant gastric mucosa: an immunohistochemical study. *J. Pathol.*, **170**, 279-283.

JOYPAUL, B.V., VOJTESEK, B., NEWMAN, E.L., HOPWOOD, D., GRANT, A., LANE, D.P. & CUSCHIERI, A. (1993*b*). Enzyme-linked immunosorbent assay for p53 in gastrointestinal malignancy: comparison with immunohistochemistry. *Histopathology* (in press).

KAPLAN, E.L. & MEIER, P. (1958). Non parametric estimation from incomplete observations. *J. Am. Stat. Assoc.*, **53**, 457-481.

KIM, J.H., TAKAHASHI, T., CHIBA, I., PARK, J.G., BIRRER, M.J., ROH, J.K., LEE, H.D., KIM, J.P., MINNA, J.D. & GAZDAR, A.F. (1991). Occurrence of p53 gene abnormalities in gastric carcinoma tumours and cell lines. *J. Natl Cancer Inst.*, **83**, 938-943.

LANE, D.P. (1993). A death in the life of p53. *Nature*, **362**, 786.

LANE, D.P. & BENCHIMOL, S. (1990). p53: oncogene or anti-oncogene? *Genes Dev.*, **4**, 1-8.

LANE, D.P. & CRAWFORD, L.V. (1979). T antigen is bound to a host protein in SV40 transformed cells. *Nature*, **278**, 261-263.

LAUREN, P. (1965). The two histological main types of gastric carcinoma: diffuse and so-called intestinal-type carcinomas: an attempt at a histological classification. *Acta Pathol. Microbiol. Scand.*, **64**, 64, 31-49.

LEE, E.T. (1980). *Statistical Methods for Survival Data Analysis*. Lifetime Learning: Belmont, CA.

LEVINE, A.J., MOMAND, J. & FINLAY, C.A. (1991). The p53 tumour suppressor gene. *Nature*, **351**, 453-456.

MCLAREN, R., KUZU, I., DUNNILL, M., HARRIS, A., LANE, D. & GATTER, K.C. (1992). The relationship of p53 immunostaining to survival in carcinoma of the lung. *Br. J. Cancer*, **66**, 735-738.

MALKIN, D., LI, F.P., STRONG, L.C., FRAUMENI, J.F., NELSON, C.E., KIM, D.H., KASSEL, J., GRYKA, M.A., BISCHOFF, F.Z., TAINSKY, M.A. & FRIEND, S.H. (1990). Germline p53 mutations in a familial syndrome of breast cancer, sarcomas and other neoplasias. *Science*, **250**, 1233-1238.

MARTIN, H.M., FILIPE, M.I., MORRIS, R.W., LANE, D.P. & SILVESTRE, F. (1992). p53 expression and prognosis in gastric carcinoma. *Int. J. Cancer*, **50**, 859-862.

MATOZAKI, T., SAKAMOTO, C., SUZUKI, T., MATSUDA, K., UCHIDA, K., NAKANO, O., WADA, K., NISHISAKI, H., KONDA, Y., NAGAO, M. & KASUGA, M. (1992). p53 gene mutations in human gastric cancer: wild-type p53 but not mutant p53 suppresses growth of human gastric cancer cells. *Cancer Res.*, **52**, 4335-4341.

MERCER, W.E., AMIN, M., SAUVE, G.J., APELLA, E., ULLRICH, S.J. & ROMANO, J.W. (1990). Wild type human p53 is antiproliferative in SV40-transformed hamster cells. *Oncogene*, **5**, 973.

MIDGLEY, C.A., FISHER, C.J., BARTEK, J., VOJTESEK, B., LANE, D.P. & BARNES, D.M. (1992). Analysis of p53 expression in human tumours: an antibody raised against human p53 expressed in *E. coli*. *J. Cell Sci.*, **101**, 183-189.

MING, S.C. (1977). Gastric carcinoma – a pathobiologic classification. *Cancer*, **39**, 2475-2485.

MIWA, K. (1984). Evaluation of the TNM classification of stomach cancer and a proposal for its rational stage-grouping. *Jpn J. Clin. Oncol.*, **14**, 385-410.

MORKVE, O. & LAERUM, O.D. (1991). Flow cytometric measurement of p53 protein expression and DNA content in paraffin-embedded tissue from bronchial carcinomas. *Cytometry*, **12**, 438-444.

NIGRO, J.M., BAKER, S.J., PREISINGER, A.C., JESSUP, J.M., HOSTTETER, R., CLEARY, K., BIGNER, S.H., DAVIDSON, N., BAYLIN, S., DEVILEE, P., GLOVER, T., COLLINS, F.S., WESTON, A., MODALI, R., HARRIS, C.C. & VOGELSTEIN, B. (1989). Mutations in the p53 gene occur in diverse human tumour types. *Nature*, **342**, 705-708.

PURDIE, C.A., O'GRADY, J., PIRIS, J., WYLLIE, A.H. & BIRD, C.C. (1991). p53 expression in colorectal tumours. *Am. J. Pathol.*, **138**, 807-813.

REIHSAUS, E., KOHLER, M., KRAISS, S., OREN, M. & MONTENARH, M. (1990). Regulation of the level of the oncoprotein p53 in non-transformed and transformed cells. *Oncogene*, **5**, 137-145.

RODRIGUES, N.R., ROWAN, A., SMITH, M.E.F., KERR, I.B., BODMER, W.F., GANNON, J.V. & LANE, D.P. (1990). p53 mutations in colorectal cancer. *Proc. Natl Acad. Sci. USA*, **87**, 7555-7559.

SANO, T., TSUJINO, T., YOSHIDA, K., NAKAYAMA, H., HARUMA, K., ITO, H., NAKAMURA, Y., NAKAJIMA, G. & TAHARA, E. (1991). Frequent loss of heterozygosity on chromosomes 1q, 5q, and 17p in human gastric carcinomas. *Cancer Res.*, **51**, 2926-2931.

SCOTT, N., SAGAR, P., STEWART, J., BLAIR, G.E., DIXON, M.F. & QUIRKE, P. (1991). p53 in colorectal cancer: clinicopathological correlation and prognostic significance. *Br. J. Cancer*, **63**, 317-319.

SUN, X.F., CARSTENSEN, J.M., ZHANG, H., STAL, O., WINGREN, S., HATSCHEK, T. & NORDENSKJOLD, B. (1992). Prognostic significance of cytoplasmic p53 oncoprotein in colorectal adenocarcinoma. *Lancet*, **340**, 1369-1373.

TAMURA, G., KIHANA, T., NOMURA, K., TERADA, M., SUGIMURA, T. & HIROHASHI, S. (1991). Detection of frequent p53 gene mutations in primary gastric cancer by cell sorting and polymerase chain reaction single-strand conformation polymorphism analysis. *Cancer Res.*, **51**, 3056-3058.

THOR, A.D., MOORE, II, D.H., EDGERTON, S.M., KAWASAKI, E.S., REIHSAUS, E., LYNCH, H.T., MARCUS, J.N., SCHWARTZ, L., CHEN, L.-C., MAYALL, B.H. & SMITH, H.S. (1992). Accumulation of p53 tumour suppressor gene protein: An independent marker of prognosis in breast cancers. *J. Natl Cancer Inst.*, **84**, 845-855.

VARLEY, J.M., BRAMMAR, W.J., LANE, D.P., SWALLOW, J.E., DOLAN, C. & WALKER, R.A. (1991). Loss of chromosome 17p13 sequences and mutation of p53 in human breast carcinomas. *Oncogene*, **6**, 413-421.

VOJTESEK, B., BARTEK, J., MIDGLEY, C.A. & LANE, D.P. (1992). An immunohistochemical analysis of the human nuclear phosphoprotein p53. *J. Immunol. Methods*, **151**, 237-244.

WATANABE, H., JASS, J.R. & SOBIN, L.H. (1990). Histological typing of gastric and oesophageal tumours. *International Histological Classification of Tumours*, pp. 20-26. Springer: London.